# Brown-shell eggs shows high incidence of blood and meat spots accompanied by unique microbial distribution patterns

**DOI:** 10.3389/fnut.2025.1561194

**Published:** 2025-03-25

**Authors:** Junfeng Wu, Yiyuan Yan, Jiahua Chen, Junying Li, Guangqi Li, Guiqin Wu, Bin Wang, Gang Zheng, Yuqin Yang, Yushuang Du, Ling Lian

**Affiliations:** ^1^State Key Laboratory of Animal Biotech Breeding, National Engineering Laboratory for Animal Breeding, China Agricultural University, Beijing, China; ^2^Beijing Engineering Research Center of Layer, Beijing, China

**Keywords:** chicken eggs, egg quality, egg contents, blood and meat spot, microbiota

## Abstract

**Introduction:**

The blood and meat spots in eggs are recognized as defects for egg quality. The frequency of blood and meat spots in brown-shell eggs is much higher than that in white-shell eggs in previous studies. However, the actual occurrence frequency and their effects on the microbial composition in eggs remain poorly understood.

**Methods:**

In this study, we examined the frequency of blood and meat spots in brown-shell and white-shell eggs, respectively, from Rhode Island Red and White Leghorn chickens at seven ages.

**Results:**

The results showed that blood and meat spots in brown-shell eggs exhibit much higher average frequency (63.99%) than that in white-shell eggs (1.37%). Furthermore, we analyzed the relationship between the presence of blood and meat spots and the microbial community distribution in the egg albumen and yolk. Briefly, we selected brown-shell eggs (*n* = 112) from Rhode Island Red, among which 51 eggs showing blood/meat spots were classified as RIR_CASE, and 61 normal eggs without blood/meat spot were classified as RIR_CON. Additional white-eggshell eggs (*n* = 124) without blood/meat spots from White Leghorn were selected as WL_CON. 16S rRNA sequencing was performed in both egg white and yolk. The results indicated that neither egg white nor yolk is sterile, with Proteobacteria identified as the dominant bacterial phyla. The microbial alpha diversity in both egg white and yolk of RIR_CASE was significantly lower compared to RIR_CON and WL_CON. Beta diversity analysis showed that the Weighted UniFrac Distance between RIR_CASE and RIR_CON in the egg yolk group was significantly larger than the distance between WL_CON and RIR_CON. It suggested that the difference of microbial diversity was mainly caused by blood and meat spots other than by chicken breeds. LEfSe analysis identified eight microbial taxa closely linked to the presence of blood and meat spots in egg white or yolk. Moreover, through the combination of random forest analysis, we identified the unique microbial biomarkers Comamonas_F and Chryseobacterium in the egg white of the RIR_CASE group.

**Discussion:**

Our study indicates that eggs with blood and meat spots occur at a higher frequency in brown-shell chickens and are accompanied by a distinct microbial community distribution.

## 1 Introduction

Eggs served as a vital, affordable source of high-quality nutrition for human, and their quality is closely linked to food safety (1). Egg quality is typically assessed in three key areas: the quality of whole egg, eggshell, and the internal contents. The whole egg quality included egg weight and shape index (2). Eggshell quality is evaluated based on color, strength, and thickness. The internal quality encompasses egg white and yolk quality, such as albumen height, haugh unit, yolk color, expect that, the presence of blood or meat spots was also an important indicator seriously affecting egg quality which was undesirable by consumers (3). The presence of blood and meat spots in eggs was first documented in 1899 (4). Blood and meat spots, also referred to as internal blood or meat inclusions, typically in three forms: blood spots, meat spots and combined blood-meat spots. Blood spots are usually found in the yolk or egg white. The blood spots showing on the yolk suggests that hemorrhaging occurred during the follicle expulsion of the egg into the ovary since blood vessel ruptured during oviposition. Blood spots in the egg white indicate that bleeding likely occurred after the egg entered the oviduct. Meat spots, which are primarily found in the egg white, usually consist of brownish deposits, necrotic tissue, or cellular debris (5, 6). As the eggs were stored for longer time, the yolk absorbs water from the egg white, diluting the blood spots and making them resemble meat spots.

The different chicken breeds showed different incidence of blood and meat spots with 18% in brown-shell eggs, compared to just 0.5% in white-shell eggs (7). Nutritional factors, a deficiency in vitamin A in the hen’s diet has also been linked to an increased incidence of blood and meat spots (8). Besides the unfavorable sensory impact on consumers, it is reported that blood and meat spots increased the risk of Salmonella contamination and decreased hatchability rates of eggs (9). Salmonella is one of the most critical foodborne pathogens globally, eggs contaminated with this bacterium could present a substantial food safety risk, endangering consumer health. The Research has shown that the primary defense mechanism against Salmonella in eggs relies on the ability of Ovotransferrin, which inhibit Salmonella growth in the albumen by restricting iron availability (10). The presence of blood and meat spots in eggs offers a significant source of iron, which may interfere with the antimicrobial activity of Ovotransferrin (11). Despite the high incidence of blood and meat spots in brown-shell eggs as well as existing potential risk to Salmonella contamination, its impacts on egg quality remain inadequately characterized.

Previous research has indicated that blood and meat spots in eggs may originate from bleeding and tissue shedding in the ovaries and oviducts during inflammatory responses (12). Recent studies further reveal that inflammatory reactions in the reproductive tissues of chickens often coincide with alterations in the composition and diversity of microbial communities (13). Since these microbial communities can be transferred from the reproductive tract to the egg contents (14), we are wondering whether microbial distribution patterns are distinct between eggs with blood and meat spots and normal eggs.

In this study, we monitored the frequency of blood and meat spots at seven ages in RIR and WL hens, and the key egg quality traits were compared between RIR and WL groups. Furthermore, we employed 16S rRNA sequencing to examine the microbial composition in brown-shell eggs with blood and meat spots, normal brown-shell eggs, and white-shell eggs, to provide the relevance between microbial communities and eggs with blood and meat spots.

## 2 Materials and methods

### 2.1 Animals

The quality of eggs from Rhode Island Red (RIR) and White Leghorn (WL) populations in the experimental farm of Beijing Huadu Yukou Poultry Breeding Co., Ltd. (Beijing, China) were observed and analyzed. The two populations were fed following standardized feeding protocols. The temperature of the house was maintained at 24 ± 1°C. The hens were kept under a controlled environment with a lighting regimen of 16 h light and 8 h dark (16 L:8D).

### 2.2 Egg quality data measurements

A total of 200 individuals from each of the population were randomly selected at each age of 22, 36, 46, 55, 63, 67, and 72 weeks. The blood and meat spot frequency of the eggs from two populations was observed. For each age point, eggs were collected over seven consecutive days for assessment of blood and meat spots. The quality of eggs collected over seven consecutive days at each time point was detected at five age points (36, 46, 55, 66, and 72 weeks). Egg weight (EW) was measured using an electronic scale with a precision of 0.01 g. Eggshell color (ESC) was quantified using a CM-2600D reflectometer (Konica Minolta, Tokyo, Japan). The eggshell strength (ESS) was then measured vertically using the Eggshell Force Gauge (Model-II, Robotmation, Tokyo, Japan). The albumen height (AH), Haugh unit (HU), and yolk color (YC), were measured using a multifunctional egg tester (EMT-7300 II, Japan Phythm Co., Ltd.). After the measurements, the egg yolk was carefully separated, and its weight (YW) was recorded using an electronic scale. Additionally, the presence of blood and meat spots was recorded to determine their occurrence frequency. The frequency of blood and meat spot occurrence was compared between the two groups at each time points. A one-way analysis of variance (ANOVA) was performed, followed by Duncan’s test to assess the differences between the two groups.

### 2.3 Experimental sample collection and design

**Figure 1:**
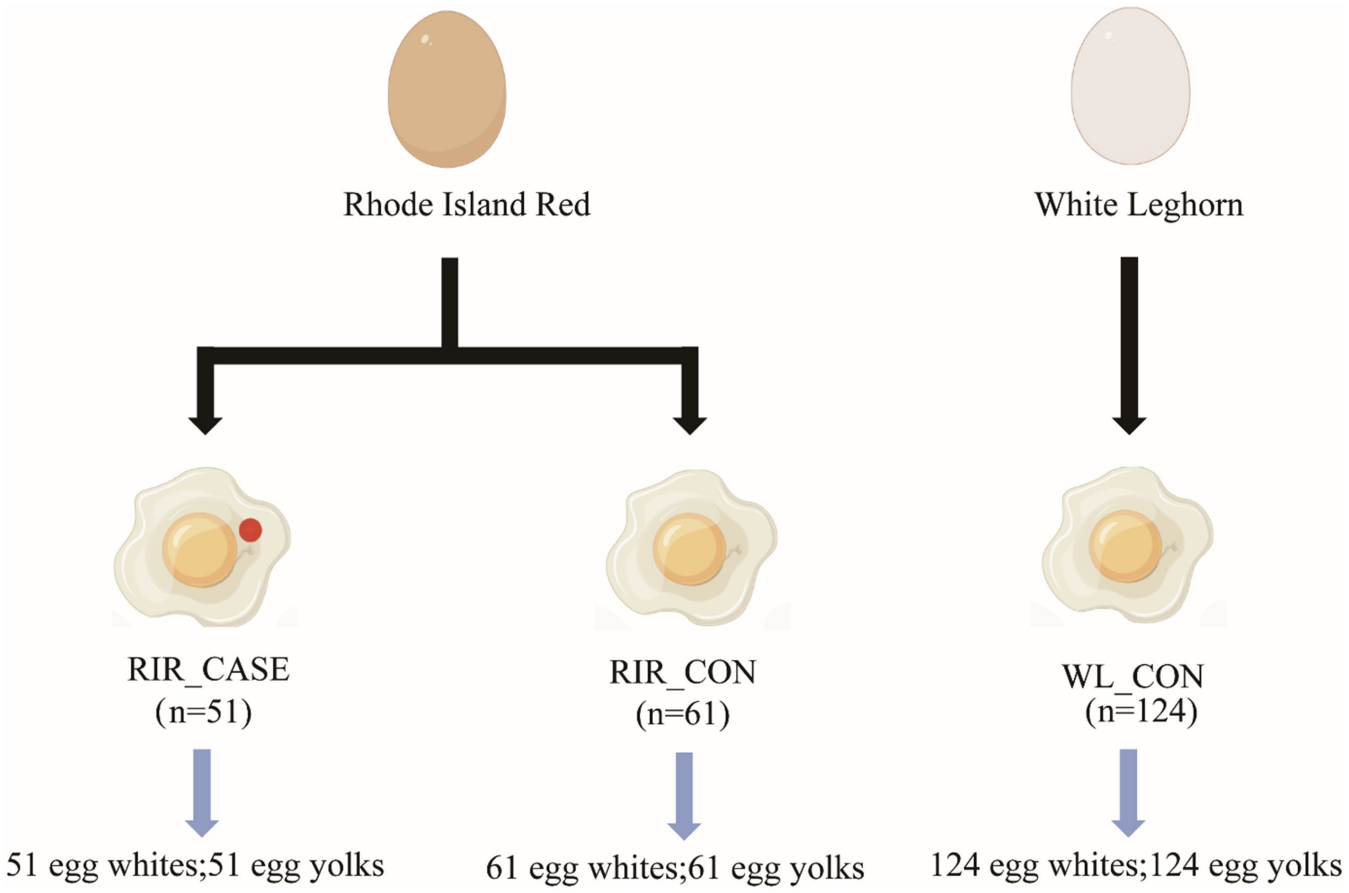
Sample collection scheme. This figure outlines the sample groups.

In order to assess the association of blood and meat spots and the microbial distribution patterns within eggs, 200 hens were selected at 66 weeks of age, including 100 Rhode Island Red hens (brown shell) and 100 White Leghorn hens (white shell). All hens were in good health, and the rearing conditions were consistent across the groups. White Leghorn hens are commonly recognized in previous studies as the breed with the lowest occurrence of blood and meat spots. Therefore, they were included in this study as a reference group. The process of sample collection is illustrated in Figure 1, we collected eggs from Rhode Island Red and White Leghorn in the three consecutive days, taking careful measures to ensure that the entire procedure was conducted under sterile conditions. In total of 51 brown-shell eggs from Rhode Island Red with blood and meat spots were classified into the RIR_CASE, 61 normal brown-shell eggs with no any blood or meat spots were classified into RIR_CON, and 124 white-shell eggs with no any blood or meat spot from White Leghorn were classified into WL_CON. Egg white (designated as “W”) and egg yolk (designated as “Y”) were separated from 236 eggs, so a total of 472 samples were collected. Fresh egg yolk and egg white were quickly frozen in liquid nitrogen and subsequently stored at −80°C for further analysis.

### 2.4 16S rRNA gene sequencing

Total genomic DNA was extracted from egg white and egg yolk of RIR_CASE, RIR_CON, and WL_CON using the OMEGA Soil DNA Kit (D5625-01) referring to the manufacturer’s protocol. The concentration and purity of the DNA were assessed using the NanoDrop 2000 spectrophotometer, and DNA quality was verified through agarose gel electrophoresis.

The V4 hypervariable region of the 16S rRNA gene was amplified using the forward primer 515F (5’-GTGCCAGCMGCCGCGGTAA-3′) and the reverse primer 806R (5’-GACTACHVGGGTWTCTAAT-3′) by PCR (15). The reaction mixture included 5 μL of 5× buffer, 5 μL of 5× GC buffer, 0.25 μL of Q5 High-Fidelity DNA Polymerase (NEB, 5 U/μl), 5 μL of dNTP (2.5 mM), 1 μL each of forward and reverse primers (10 μM), 2 μL of DNA template, and 8.75 μL of ddH2O. The PCR conditions were: 98°C for 2 min, followed by 25–30 cycles of 98°C for 15 s, 55°C for 30 s, and 72°C for 30 s, with a final extension at 72°C for 5 min, and the reaction held at 10°C. PCR products were purified with Vazyme VAHTSTM DNA Clean Beads (Vazyme, Nanjing, China) and quantified using the Quant-iT PicoGreen dsDNA Assay Kit (Invitrogen, Carlsbad, CA, United States). Following quantification, barcoded V4 amplicons were pooled and sequenced using the Illumina NovaSeq platform with the NovaSeq 6,000 SP reagent kit (Shanghai Pasono Biotech Co., Ltd., Shanghai, China), producing paired-end reads of 250 base pairs (bp).

### 2.5 Bioinformatic analysis

Microbial community analysis was conducted using QIIME2 version 2022.11 (16) following the official guidelines. Raw sequencing data was processed using the demux plugin for demultiplexing, the cutadapt plugin for primer trimming, and the DADA2 (17) plugin for quality filtering, denoising, merging, and removal of chimeric sequences. Sequences were clustered at 100% similarity (18) to generate Amplicon Sequence Variants (ASVs) and abundance tables. ASVs with abundance values below 0.001% of the total sequencing reads across all samples were excluded (19, 20), resulting in 38,599 retained ASVs. Taxonomic classification and relative abundance were calculated at the domain, phylum, class, order, family, genus, and species levels.

Alpha diversity was assessed using QIIME2, with the Shannon and Simpson indices employed to characterize the diversity levels between groups (21). Statistical significance was determined using the Kruskal-Wallis test and Dunn’s test for *post hoc* analysis. For beta diversity, Bray–Curtis dissimilarity was utilized to investigate microbial community structure changes between samples. Weighted UniFrac distances were calculated to compare beta diversity across groups, and differences were evaluated using the ANOSIM (Analysis of Similarities) test (22). The functional capabilities of the microbial communities were predicted using PICRUSt2 software (23) referencing the MetaCyc^1^ and KEGG^2^ databases. Linear Discriminant Analysis Effect Size (LEfSe) was employed to identify significantly different microbial taxa among groups (24). The Kruskal-Wallis test identified taxa with significant differences, and the group with the highest abundance was selected for further differential testing. Wilcoxon tests assessed the significance of inter-group differences, identifying significantly different species. LDA analysis was used to estimate the effect size of each differential species on group differences, with a threshold of 4 applied to classify marker species. To further refine the identification of key microbial communities within the different groups, we utilized the random forest decision tree algorithm (25), the model underwent ten-fold cross-validation, and the differential microbial genera identified were ranked based on their importance.

## 3 Results

### 3.1 Occurrence frequency of blood and meat spots across different groups

**Figure 2:**
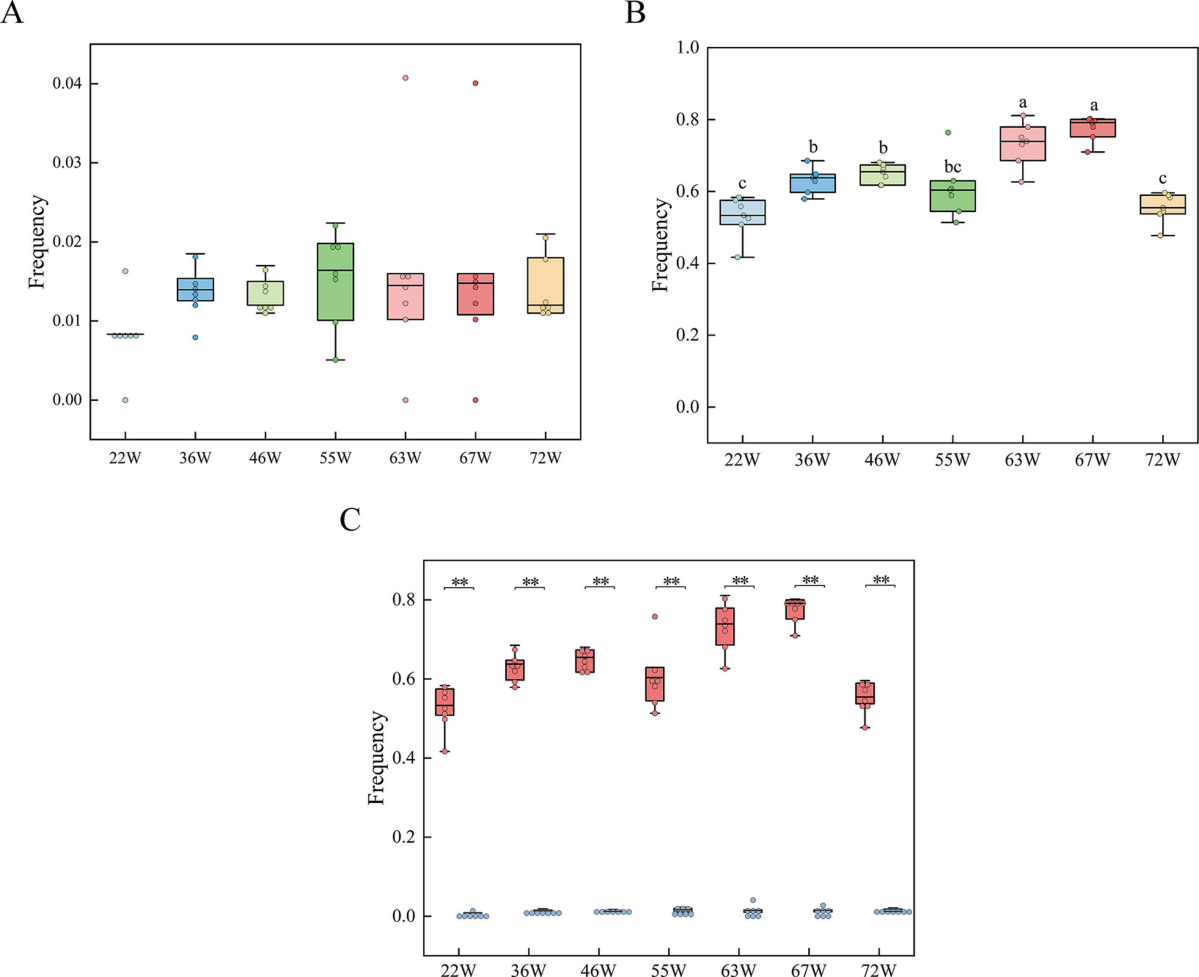
Frequency tracking of blood and meat spots in RIR and WL populations throughout the laying cycle. **(A)** Blood and meat spot frequencies in the RIR population over the entire laying period, with distinct letter annotations denoting statistically significant differences among groups. **(B)** Blood and meat spot frequencies in the WL population across the laying period. **(C)** Comparative analysis of blood and meat spot frequencies between RIR and WL populations during the laying cycle. ** *p* < 0.01.

In this study, we analyzed the frequency of blood and meat spots in the eggs of RIR and WL chickens at different ages during their laying period. For the Rhode Island Red group, the incidence of eggs with blood and meat spots gradually increased from 22 weeks of age, peaked at 63 and 67 weeks, and then sharply declined at 72 weeks. The average occurrence of blood and meat spots averaged 63.99% (Figure 2A; Supplementary Table 2). In contrast, in the White Leghorn group, the frequency of eggs with blood and meat spots remained very low throughout the laying period, with an average incidence of 1.37% (Figure 2B; Supplementary Table 2). The frequency of eggs with blood and meat spots in Rhode Island Red chickens was significantly higher than in White Leghorn chickens (Figure 2C; Supplementary Table 2), consistent with previous reports that White Leghorns have an extremely low incidence of blood and meat spots.

Moreover, the seven egg quality traits (albumen height, haugh unit, eggshell strength, egg weight, yolk color, yolk weight, egg production, and eggshell color) were similar between Rhode Island Red and White Leghorn chickens at all ages (Supplementary Table 1). For example, the albumen height and haugh unit phenotypes in both populations gradually increased after 36 W until peaking at 66 W, with both showing a decreasing trend at 72 W. These results suggested that blood and meat spots occurred more frequently in brown-shell eggs, which did not affect the egg quality traits.

### 3.2 Descriptive statistics for sequencing outputs

**Figure 3:**
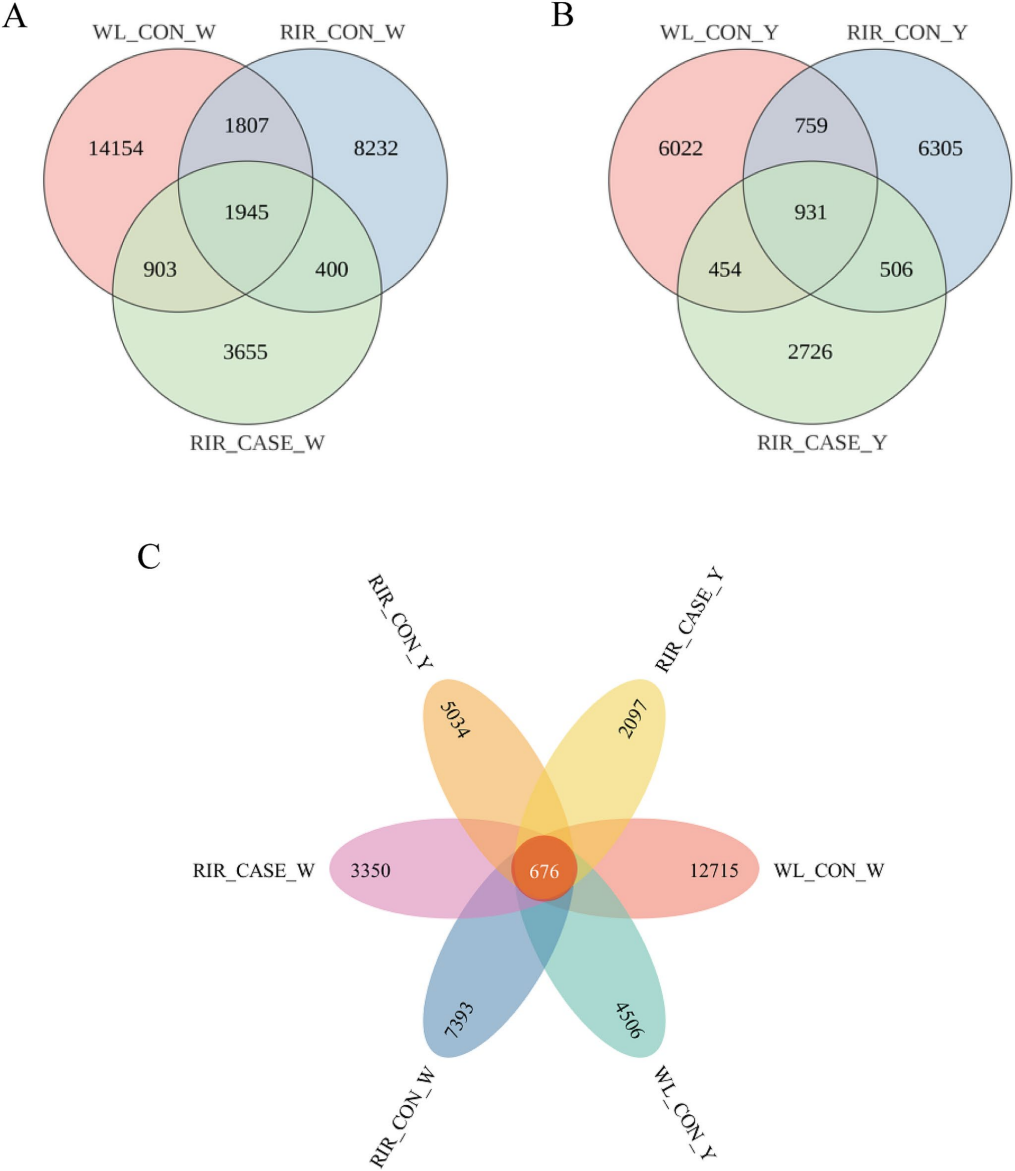
Venn diagrams illustrating the shared ASVs among the groups. **(A)** The unique and shared ASVs in albumen among the three groups. **(B)** The unique and shared ASVs in yolk among the three groups. **(C)** The unique and common ASVs in egg albumen and yolk among three groups.

Both egg white and yolk were detected. The egg white and yolk of RIR_CASE, RIR_CON, WL_CON groups was designated as RIR_CASE_W, RIR_CON_W, and WL_CON_W, as well as RIR_CASE_Y, RIR_CON_Y, and WL_CON_Y, respectively. After quality control, a total of 46,456,738 reads were obtained, with an average of 98,540 reads per sample (Supplementary Table 3). These reads were used to create a feature table comprising 39,151 ASVs. The ASVs were subsequently categorized into 84 phyla, 218 classes, 609 orders, 1,277 families, 3,698 genera, and 6,902 species. A Venn diagram was used to depict the number of shared ASVs in egg white and yolk across the three groups. Specifically, in egg white, the three groups shared 6.10% of ASVs (1,945) (Figure 3A), while in the yolk, three groups shared 5.26% of ASVs (931) (Figure 3B). Combining egg white and yolk, there was total of ASVs (676) which account for 1.73% were common to all groups (Figure 3C).

### 3.3 Diversity of microbiota in egg contents

**Figure 4:**
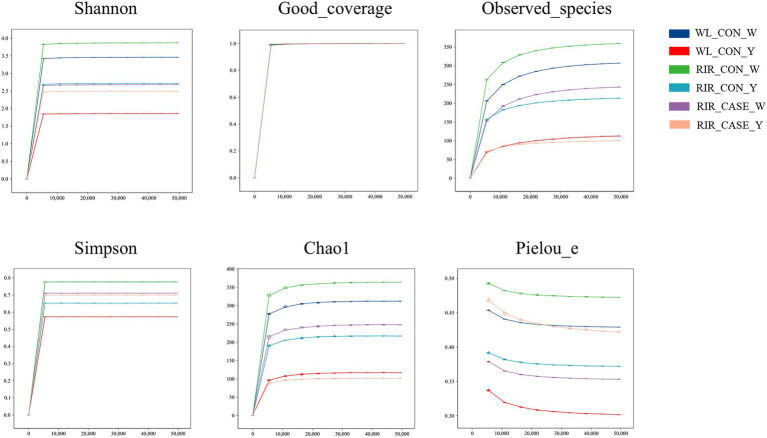
Rarefaction curves for alpha diversity. Rarefaction curve analysis of alpha diversity indices in egg white and yolk among three groups. The x-axis represents the rarefaction depth, while the y-axis shows the median values of alpha diversity indices from 10 calculations, displayed alongside box plots. The degree of curve flatness indicates the effect of sequencing depth on observed sample diversity.

**Figure 5:**
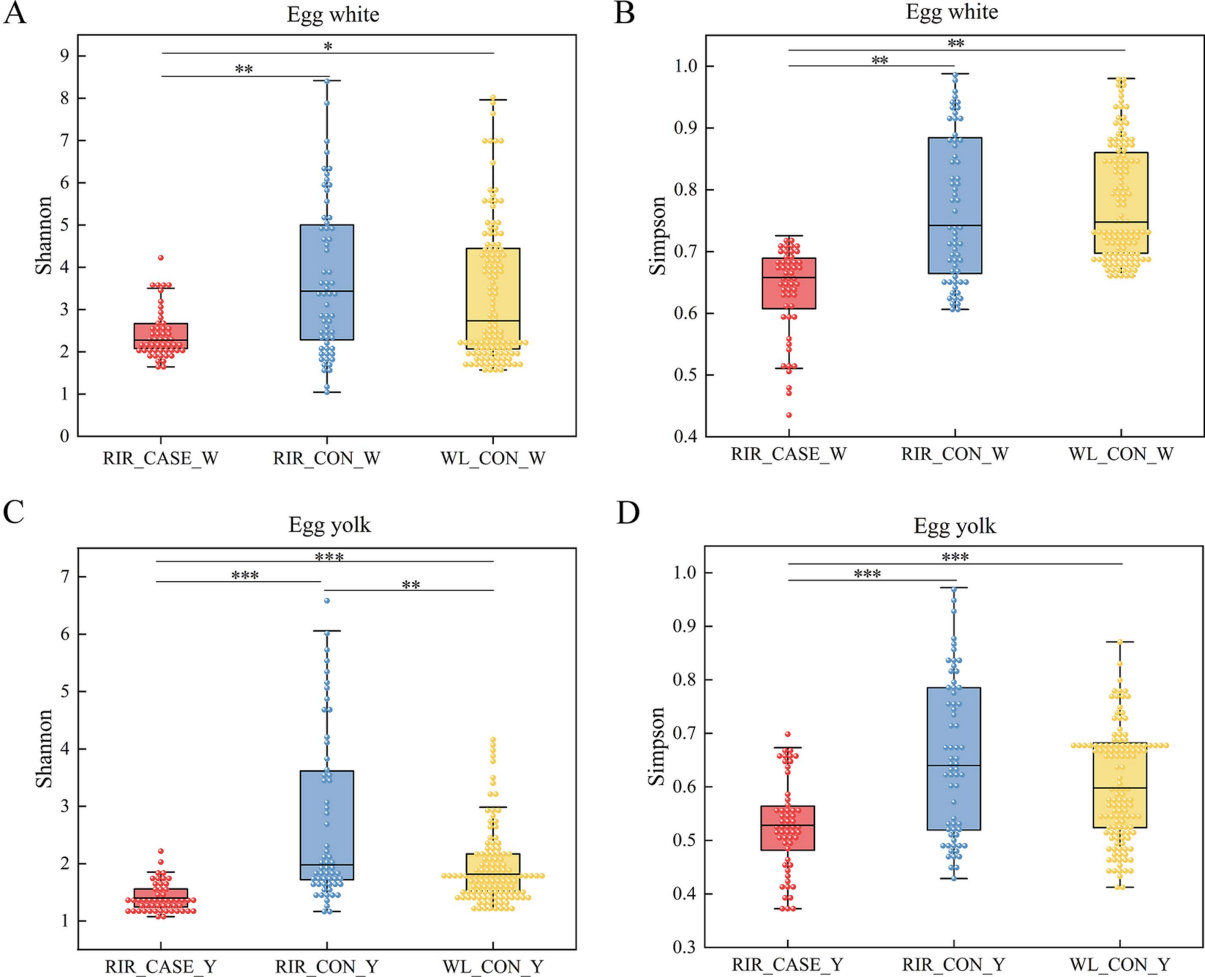
Significance tests for alpha diversity. **(A)** Shannon index comparison among the three groups in the egg albumen. The x-axis shows group labels, and the y-axis shows the corresponding alpha diversity index. **(B)** Simpson index comparison in the egg white among the three groups. The x-axis represents shows group labels, and the y-axis shows the corresponding alpha diversity index. **(C)** Shannon index comparison in the egg yolk among the three groups. The x-axis shows group labels, and the y-axis shows the corresponding alpha diversity index. **(D)** Simpson index comparison in the egg yolk among the three groups. The x-axis shows group labels, and the y-axis shows the corresponding alpha diversity index. All values were shown as the mean ± SD, * *p* < 0.05, ** *p* < 0.01, *** *p* < 0.001.

We assessed the alpha diversity of microbial communities among three groups. Rarefaction curve analysis confirmed that the sequencing depth was sufficient, with all groups reaching saturation, ensuring comprehensive coverage for analysis (Figure 4). The Shannon and Simpson indices revealed that the microbial diversity in both egg white (Figures 5A,B) and yolk (Figures 5C,D) of the RIR_CASE group was significantly lower compared to the other two groups, with no significant difference observed between RIR_CON and WL_CON. It indicated that blood/meat spots were closely associated with lower microbial diversity, which also revealed that the hens laid abnormal egg were suffering microbial imbalance.

**Figure 6:**
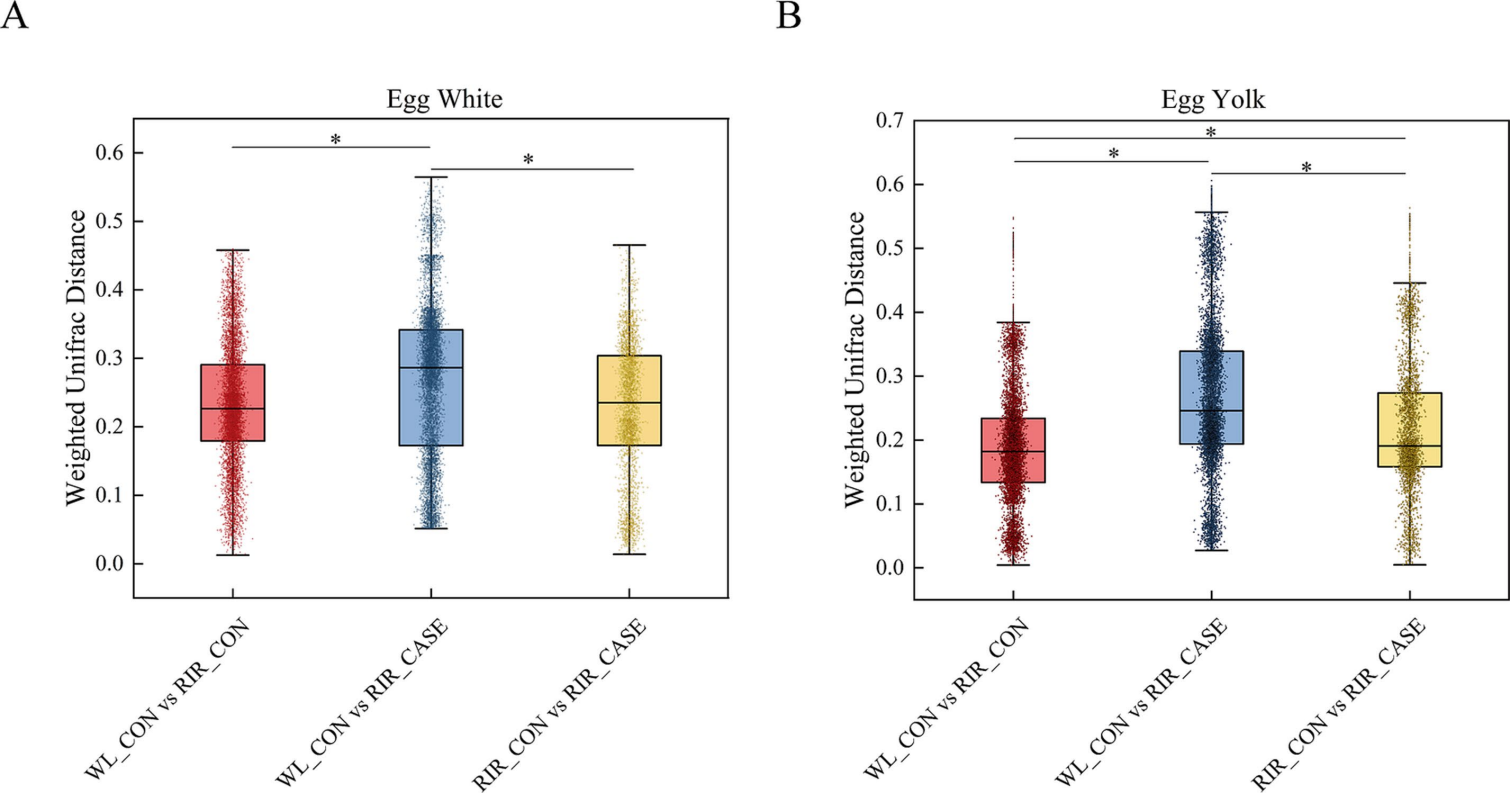
Variation in microbial Weighted UniFrac distances across three groups. **(A)** Comparison of microbial Weighted UniFrac distances in egg white among there groups. **(B)** Comparison of microbial Weighted UniFrac distances among in egg yolk among there groups. * *p* < 0.05.

In the egg white, the Weighted UniFrac distance in WL_CON vs. RIR_CASE was significantly larger than that in RIR_CASE vs. RIR_CON and WL_CON vs. RIR_CON (Figure 6A). In the egg yolk, a similar pattern was observed, where the Weighted UniFrac distance in WL_CON vs. RIR_CASE was significantly larger than the distance in WL_CON vs. RIR_CON and RIR_CASE vs. RIR_CON (Figure 6B). Notably, the Weighted UniFrac distance in RIR_CASE vs. RIR_CON was significantly larger than the distance in WL_CON vs. RIR_CON (Figure 6B), indicating that the microbial distribution difference caused by blood and meat spots was even greater than that caused by chicken breeds.

### 3.4 Compositional structure of microorganisms in egg contents

**Figure 7:**
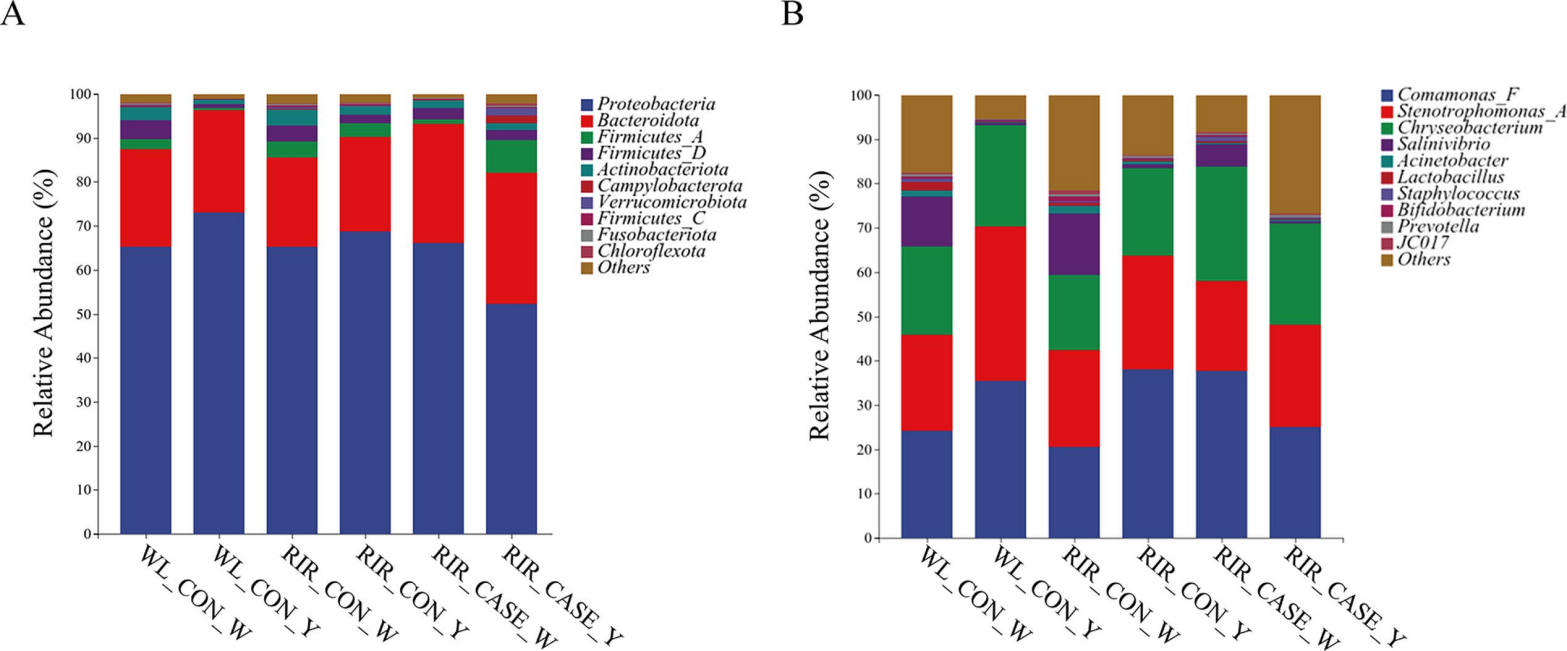
Microbial community composition in egg white and yolk from three groups. **(A)** Abundance of dominant microbial phyla in egg white and yolk from three groups; **(B)** Abundance of dominant microbial genera in egg white and yolk from three groups.

The dominant bacterial phyla in the egg contents are *Proteobacteria, Bacteroidetes*, *Firmicutes* and *Actinobacteria*. The most predominant phylum, *Proteobacteria*, accounts for 65.18 and 68.92% of the microbial community in egg white and egg yolk, respectively (Figure 7A; Supplementary Table 4). *Bacteroidetes*, the second most abundant phylum, accounts for 21.40 and 23.97% in egg white and egg yolk, respectively (Figure 7A; Supplementary Table 4). Notably, in RIR_CASE, the abundance of *Proteobacteria* in the egg white was higher than that in the yolk, while an opposite trend is observed in the other two groups. Furthermore, *Bacteroidetes* is more prevalent in both egg white (27.08%) and yolk (29.75%) of RIR_CASE groups compared to the WL_CON (22.28 and 23.37%) and RIR_CON groups (20.15 and 21.50%) (Figure 7A; Supplementary Table 4).

At the genus level, *Comamonas_F*, *Stenotrophomonas_A* and *Chryseobacterium* exhibit high relative abundance in both egg white and egg yolk samples (Figure 7B). Interestingly, *Comamonas_F* and *Chryseobacterium* show higher abundances in the egg white than that in the yolk for RIR_CASE group (Figure 7B; Supplementary Table 5). Conversely, these genera display higher abundance in the egg yolk than that in egg white in the WL_CON and RIR_CON groups (Figure 7B; Supplementary Table 5).

### 3.5 Functional annotation of microorganisms

**Figure 8:**
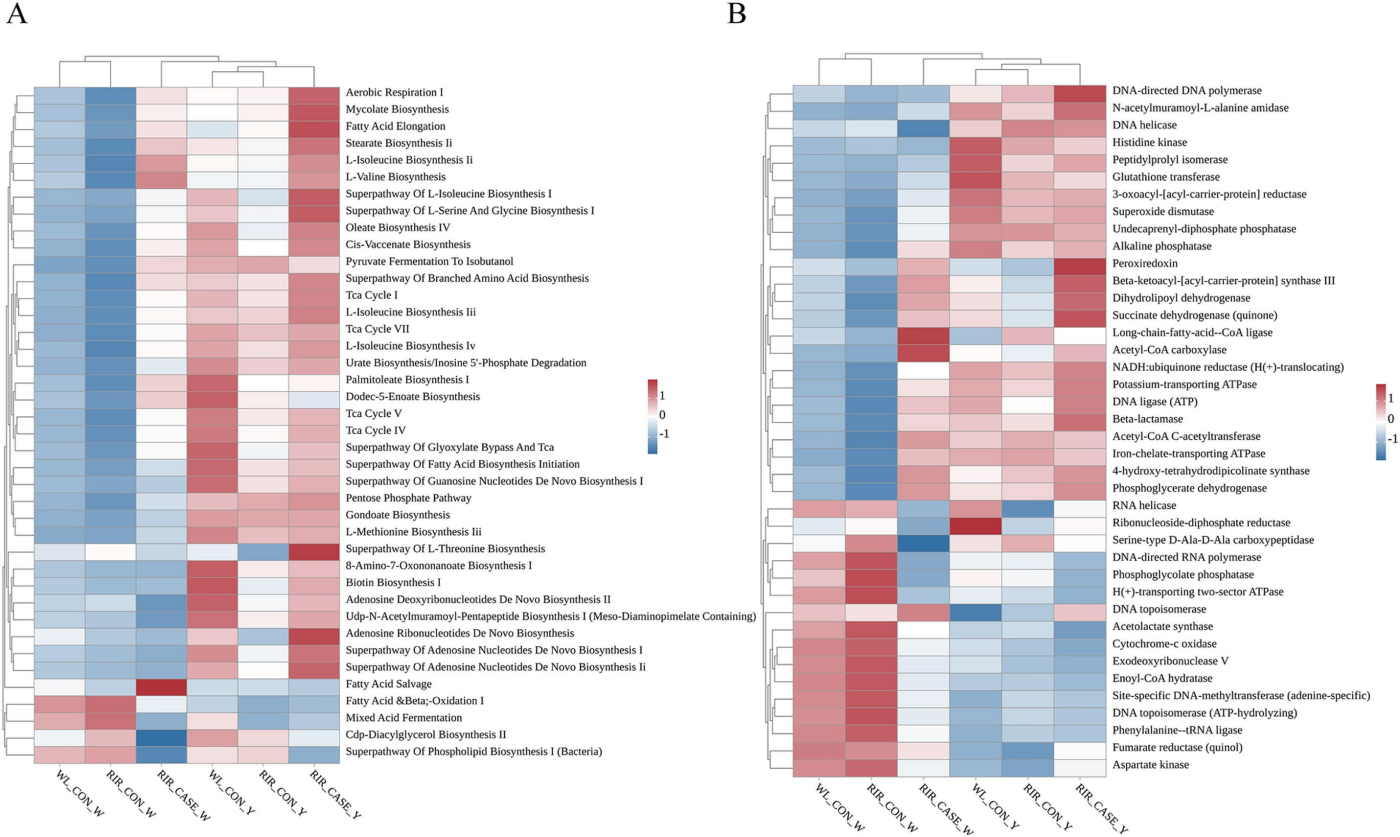
Heatmaps illustrating KEGG pathway analysis and MetaCyc metabolic pathway analysis of microbial communities enriched in. **(A)** Heatmap illustrating the KEGG functional pathways in the different groups. **(B)** Heatmap showing the MetaCyc metabolic pathways in the different groups.

Potential functions and metabolic pathways of the microbial communities in egg contents were predicted using PICRUSt2. We focused on the top 40 KEGG and MetaCyc pathways based on their relative microbial abundances (Figures 8A,B). In the egg white, KEGG analysis revealed that microbes with high abundance in WL_CON and RIR_CON groups were enriched in pathways of fatty acid oxidation, glycerol biosynthesis, and the superpathway of phospholipid biosynthesis. While, the microbes with high abundance in the RIR_CASE enriched in the processes such as aerobic respiration and bacterial biosynthesis, including amino acid synthesis pathways (Figure 8A). Similarly, in the yolk group, KEGG analysis showed that the RIR_CASE group differed significantly from the WL_CON and RIR_CON groups in the abundance of a number of functional pathways, including palmitoleic acid biosynthesis I, fatty acid elongation, and L-threonine biosynthesis superpathway (Figure 8A).

MetaCyc pathway analysis indicated that microbes more prevalent in egg white of WL_CON and RIR_CON groups were enriched in acetolactate synthase and cytochrome c oxidase pathways (Figure 8B). While, microbial communities with higher relative abundance in the RIR_CASE group was related to enzyme metabolic pathways, including peroxidase and alkaline phosphatase. In the yolk, the metabolic pathways enriched in the microbial community of the RIR_CASE group differed significantly from those of the WL_CON and RIR_CON groups, including pathways such as RNA helicase and DNA topoisomerase (Figure 8B). Overall, these findings highlighted the microbial communities of the RIR_CASE group, especially for egg white, enriched in different substantial functional and metabolic pathways, compared to the other two groups.

### 3.6 Identification of differential microorganisms

**Figure 9:**
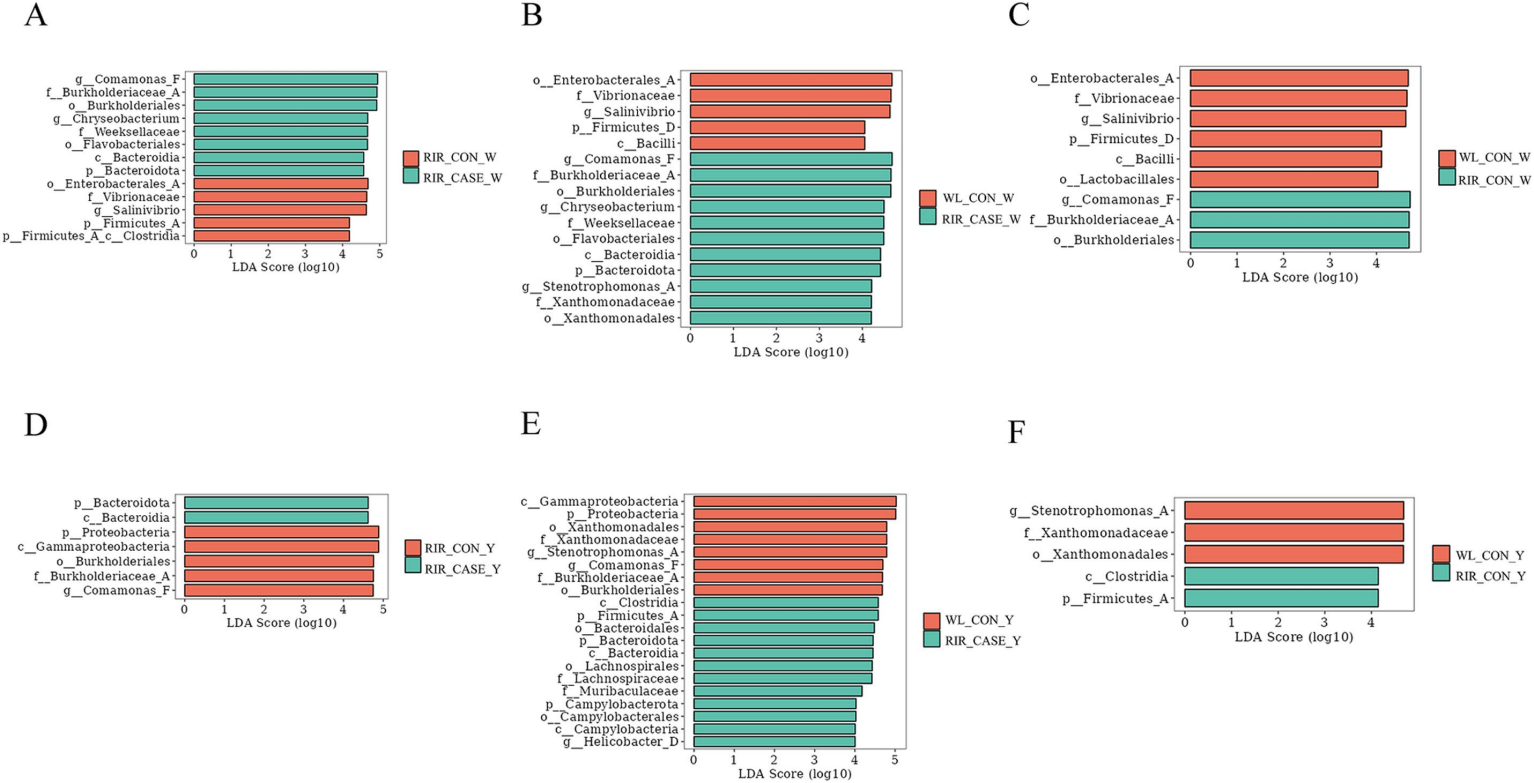
LEfSe analysis of differential microbial communities in egg white and egg yolk groups. **(A)** LEfSe analysis of biomarker species between the WL_CON _W and RIR_CON_W groups. **(B)** LEfSe analysis comparing the microbial biomarkers in the WL_CON_W and RIR_CASE_W groups. **(C)** Differential microbial biomarker analysis between the RIR_CON_W and RIR_CASE_W groups using LEfSe. **(D)** Comparison of microbial biomarkers between the WL_CON_Y and RIR_CON_Y groups through LEfSe. **(E)** LEfSe-based differential biomarker analysis between the WL_CON_W and RIR_CASE_W groups. **(F)** Differential microbial biomarker analysis between the RIR_CON_Y and RIR_CASE_Y groups using LEfSe.

**Figure 10:**
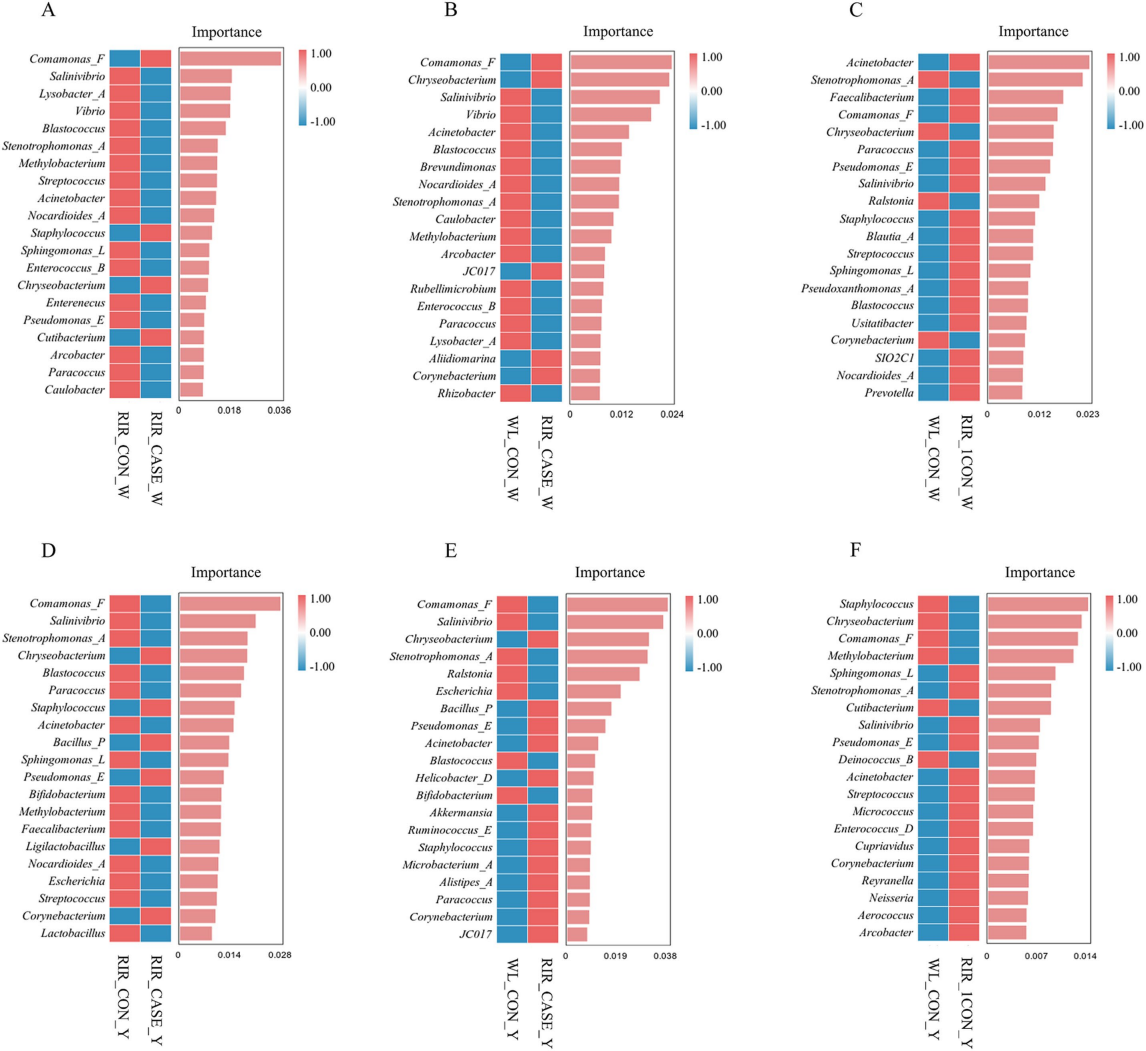
Random forest analysis of differential microbial communities in egg white and egg yolk groups. **(A)** Random forest analysis of biomarker species between the RIR_CON_W and RIR_CASE_W groups. **(B)** Random forest analysis comparing the microbial biomarkers in the WL_CON_W and RIR_CASE_W groups. **(C)** Differential microbial biomarkers identified through random forest analysis. Between the WL_CON _W and RIR_CON_W groups using LEfSe. **(D)** Random forest analysis comparing the microbial biomarkers between the RIR_CON_Y and RIR_CASE_Y groups. **(E)** Random forest-based differential biomarker analysis between the WL_CON_W and RIR_CASE_W groups. **(F)** Differential microbial biomarker analysis between the WL_CON_Y and RIR_CON_Y groups using random forest.

To identify microbial taxa with significant differential abundance among three groups, LEfSe analysis and random forest analysis was employed at the genus level. In this study, we proposed that the comparison between the RIR_CON group and the RIR_CASE group reveals microbial markers associated with blood spot formation (Figures 9A,D, 10A,D). Meanwhile, the comparison between the WL_CON group and the RIR_CON group identifies microbial biomarkers due to species difference (Figures 9C,F, 10C,F). Furthermore, the comparison between the WL_CON group and the RIR_CASE group uncovers biomarkers reflecting both species differences and blood spot occurrence (Figures 9B,E, 10B,E).

Our analysis revealed that, in both the egg white and yolk, the number of biomarkers identified by LEfSe analysis was highest between the WL_CON and RIR_CASE groups (Figures 9B,E). Interestingly, the biomarker number between the RIR_CASE and RIR_CON groups (Figures 9A,D) was significantly more than that between the RIR_CON and WL_CON groups (Figures 9C,F) in both egg white and yolk. These findings further suggested that the microbial community variations resulted from the presence of blood and meat spots are more pronounced than that from different chicken breeds (Figure 9).

To further characterize the key microbial differences between the groups in both egg white and yolk, we conducted a random forest analysis. In the egg white, we found that, compared to the RIR_CON group, the RIR_CASE group exhibited unique microbial biomarkers, including Comamonas_F, Staphylococcus_vlococeus, Chryseobacterium, and Cutibacterium (Figure 10A). Notably, Comamonas_F and Chryseobacterium were also identified as intersecting biomarkers in the LEfSe analysis. Furthermore, the yolk analysis revealed that, in comparison to the RIR_CON group, the RIR_CASE group harbored distinct microbial communities, characterized by biomarkers such as Chryseobacterium, Staphylococcus, Bacillus_P, Pseudomonas_E, Ligilactobacillus, and Corynebacterium (Figure 10D), however, these biomarkers were not overlapped with the biomarkers identified in the LEfSe analysis.

## 4 Discussion

Eggs are an affordable and essential source of protein in the human diet. As a staple food, their consumption remains consistently high. Many previous studies focused on the quality of egg white and yolk, but there were just a few researches concentrating on blood and meat spots. This study demonstrates that the frequency of blood and meat spots is significantly higher in RIR compared to WL. In RIR chickens, the frequency of blood and meat spots increases with age, whereas WL chickens consistently show an extremely low incidence of blood and meat spots, which is consistent with previous reports (12). Furthermore, the seven egg quality characteristics of brown-shell eggs produced by the RIR chickens are comparable to those of white-shell eggs produced by the WL chickens. It was consistent with recent study about egg quality traits in both breeds which showed egg quality traits were found to be similar between RIR and WL (26). These findings suggest that the presence of blood and meat spots does not significantly affect the core quality attributes of eggs.

The most obvious and visible difference between brown egg and white egg is eggshell color. The pigment of brown-shelled eggs is primarily protoporphyrin. Early studies suggested that it originates from normal erythrocyte destruction in the mucous layer of the oviduct and is transported by specific cells into uterine epithelium during the calcification stage, depositing the pigment into the eggshell (27). Later studies, however, showed that protoporphyrin was synthesized in the shell gland (28, 29), and they found the Rhode Island Red has higher activity of several porphyrin biochemical enzymes in the shell gland compared to the White Leghorn (29). All of the studies above clarified that pigment was released from oviduct to eggshell. Pigment deposition during egg formation represents a key distinction between brown-shelled and white-shelled chickens. Consequently, we hypothesize that the process of brown-shelled egg formation may elevate the risk of damage and inflammation in the oviduct. Given that the oviduct is the primary site for albumen production, this could explain the higher incidence of blood and meat spots observed in brown-shelled eggs relative to their white-shelled counterparts.

Our research confirmed microorganisms existed in both the egg white and yolk, indicating that the egg formation process is not sterile, which aligns with previous study (14). Moreover, we found that microbial communities in egg white and yolk of brown-shell eggs with blood and meat spots was different from those in white-shell and normal brown-shell eggs. The average frequency of blood and meat spot eggs observed in brown-shelled chickens in this study was 63.99%, substantially higher than the 18% reported in earlier research (12). This difference may be attributed to the stricter evaluation criteria adopted in this study, which classified even the tiny red or brown blood and meat spots in the egg content as blood and meat spots.

We found that primary dominant microbial phyla in egg contents were also consistent with previous study (30). The *Proteobacteria* are typically regarded as opportunistic pathogens detrimental to human health. Nevertheless, some research suggests that certain members of this phylum may also contribute beneficially to maintaining the host’s health (31). Studies on eggs have shown that the majority of microorganisms in *Proteobacteria* are facultative anaerobes, which help to stabilize the egg’s pH and decreased its redox potential (32). Additionally, certain facultative bacterial genera within *Proteobacteria* can facilitate the colonization of other facultative anaerobes. Previous research on microbiota of chicken reproductive tract has demonstrated that *Firmicutes*, *Proteobacteria*, *Actinobacteria*, *Clostridia,* and *Bacteroidetes* are the predominant phyla (15). Moreover, another study identified a total of 21 shared genera across the host’s oviduct microbiota, egg content microbiota, eggshell microbiota, and embryonic intestinal microbiota, highlighting the similarity resulting from vertical microbial transmission (33), which means the egg white and yolk acquire a portion of the host’s microbial flora during their formation.

Recent studies on the reproductive tract microbiome have uncovered a strong connection between specific microbial communities in the female uterus and host reproductive health (34–36). Research in humans, mice, and other model organisms has demonstrated that shifts in the uterine microbiome are closely associated with reproductive disorders and changes in host physiology (37–39). Hence, we proposed that changes in the microbial composition in egg contents might indicate reproductive system dysfunctions or physiological declines in the laying hens.

In both egg white and egg yolk studies, the alpha diversity of brown-shell eggs with blood and meat spots was markedly lower compared to that of white-shell eggs and normal brown-shell eggs, suggesting a more simplified microbial composition. Previous research on the microbiota of chicken reproductive systems and eggs revealed that older hens exhibit a significantly lower Shannon index in their oviduct microbiota than younger hens, indicating microbial imbalance (13). Moreover, older hens often present inflammatory responses in late-stage egg production, and the uterine microenvironment shows heightened innate immune responses and elevated inflammation markers. This correlation between microbiota imbalance and increased innate immunity in pathological hosts underscores the role of dysbiosis in triggering inflammatory response (40, 41). Blood and meat spots in eggs are generally linked to small hemorrhages or tissue damage within the chicken’s reproductive tract (such as the oviduct), which are part of an inflammatory process, suggesting local immune system activation (12). Research conducted by Mervi also reported that presence of blood spots is strongly linked to inflammatory responses within the reproductive system (12). Following such an inflammatory event, the colonization of the entire reproductive tract microbiome is affected, which, in turn, leads to changes in the microbial composition of the egg (42–44). Given the strong similarity between the microbiota of eggs and the reproductive tract, we proposed that microbial dysbiosis in the reproductive microenvironment of hens leads to overactivation of the innate immune system, further disrupting microbial equilibrium. This, in turn, makes tissues more susceptible to damage or recurrent inflammatory episodes, such as hemorrhaging, and finally results in eggs with blood and meat spots.

In this study, we investigated the functional pathways and metabolic processes of the microbiota present in egg contents, utilizing the KEGG and MetaCyc databases. Whether it is the egg white or the egg yolk, the microbial communities in white-shell eggs and normal brown-shell eggs share substantial similarities in terms of functional pathways and metabolic processes., interestingly, within the brown-shell chicken group, RIR_CON and RIR_CASE displayed more pronounced differences in these pathways and metabolic processes.

The LEfSe analysis conducted in this study suggests that eggs with blood spots showing higher abundance of certain microbial communities, including microbial communities from the genus Chryseobacterium, family Weeksellaceae, order Flavobacteriales, and order Burkholderiales, which was considered to be primarily associated with pathogenicity. All these bacteria are aerobic, heat-sensitive, and resistant to various antibiotics (45–47). Random forest analysis further identified two unique microbial biomarkers in the egg white of the RIR_CASE group: Comamonas_F and Chryseobacterium. Comamonas_F has been widely recognized for its role in degrading organic waste, during which it eliminates pathogens and impurities (48). In this study, Comamonas_F was found to be a specific biomarker in the RIR_CASE_W group. We hypothesized that the occurrence of blood and meat spots introduces impurities and potential pathogens into the egg. In such an environment, Comamonas_F, which is involved in metabolic processes and degradation, is upregulated. Conversely, Chryseobacterium, a known opportunistic pathogen, has been associated with a variety of clinical infections, including pneumonia, peritonitis, wound infections, bacteremia, and cellulitis (49). Thus, the presence of Chryseobacterium in the RIR_CASE_W group could represent a potential infection risk. Collectively, we hypothesized the cause for blood and meat spots was that dysbiosis in the microbial environment of Rhode Island red laying eggs with blood and meat spots suppressed environmentally sensitive beneficial bacteria, giving opportunistic pathogens a greater chance to proliferate and leading to an inflammatory injury of ovary and oviduct with blood, or necrotic tissue, or cellular debris detached and dropped into eggs.

## 5 Conclusion

In summary, our findings clearly indicated that the occurrence of blood and meat spots is significantly higher in RIR chickens compared to WL chickens, reaching a peak in the later stages of egg production. Eggs with blood and meat spots exhibit lower microbial diversity and a more uniform microbial composition, with the impact of blood and meat spots even surpassing inter-breed differences. This study, for the first time, compared a detailed profile of the microbial composition between normal and blood/meat-spot eggs, giving an explicit relevance of microbial variation with blood and meat spots. These findings provide new insights for identifying factors associated with the occurrence of blood and meat spots.

## Data Availability

The sequence data has been deposited to the National Center for Biotechnology Information (NCBI) with the accession code PRJNA1229787. All sequencing data can be retrieved at this link: https://www.ncbi.nlm.nih.gov/bioproject/PRJNA1229787.

## Footnotes

^1^ https://metacyc.org/

^2^ https://www.kegg.jp/
