# Correction: Brown-shell eggs shows high incidence of blood and meat spots accompanied by unique microbial distribution patterns

**DOI:** 10.3389/fnut.2025.1652275

**Published:** 2025-07-08

**Authors:** Frontiers Production Office

**Affiliations:** Frontiers Media SA, Lausanne, Switzerland

**Keywords:** chicken eggs, egg quality, Egg contents, blood and meat spot, microbiota

In the published article, there was an error in Figure 1 as published. The position of labels “WL_CON (*n* = 124)” and “RIR_CASE (*n* = 51)” in Figure 1 were erroneously reversed. The corrected Figure 1 and its caption appear below.

**Figure 1:**
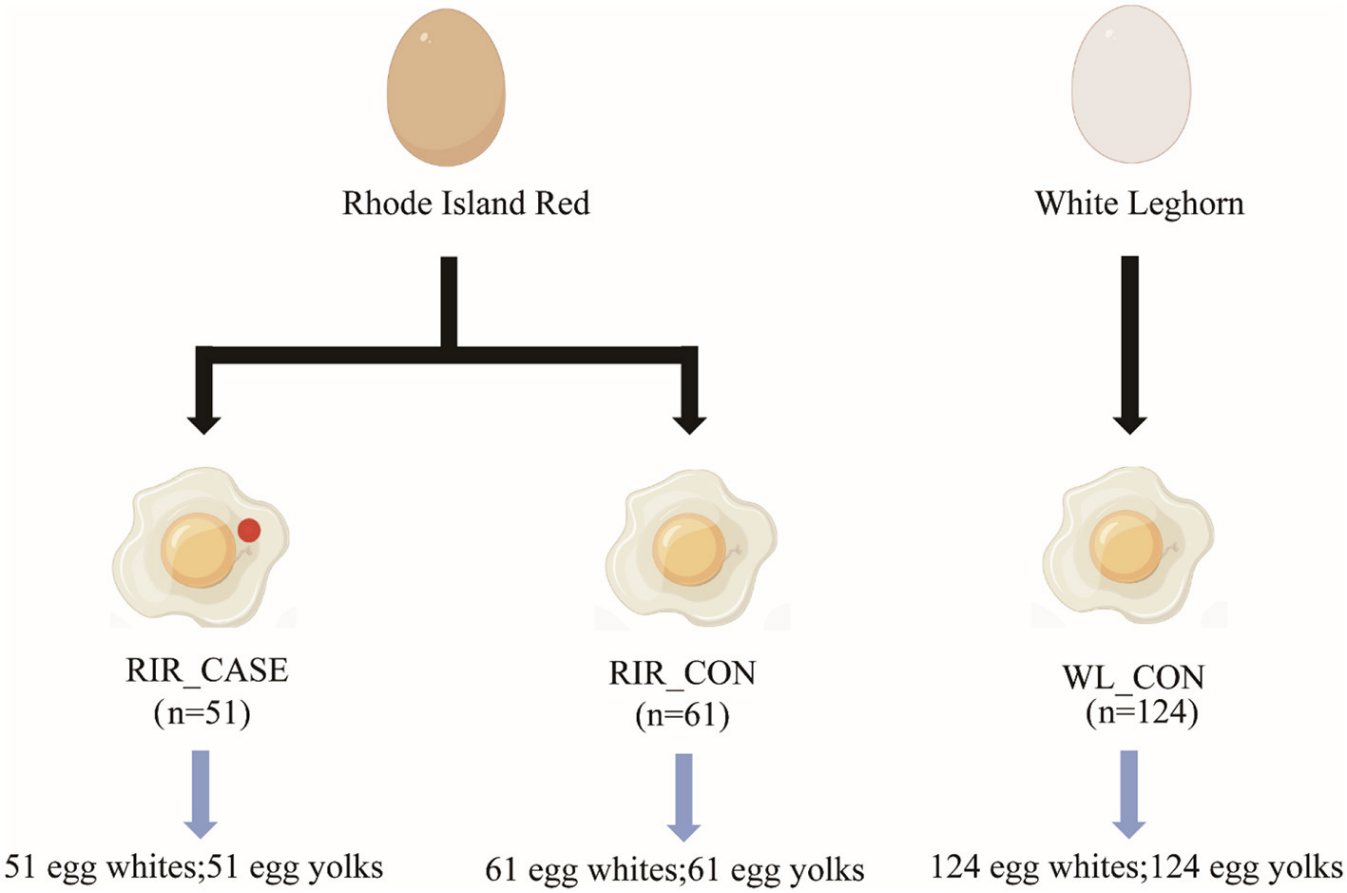
Sample collection scheme. This figure outlines the sample groups.

The original version of this article has been updated.

